# Observations Relative to the Treatment by Sir William Adams of the Ophthalmic Cases of the Army

**Published:** 1818-06

**Authors:** 


*Observations relative to the Treatment by Sir William Adams of the Ophthalmic Cases of the Army.* By JOHN VETCH, M.D. Physician to the Forces, &c. London, 1818.

SIR WM. ADAMS must either be "much sinned against, or much sinning," to draw such a cloud of enemies around him from every quarter of the compass. It is true, that he may quote the couplet of the poet—

> All human virtue, to its latest breath,
> Finds *Envy* never conquer'd but by death.

Yet, if we closely examine the medical history of *our own times,* we shall be constrained to acknowledge (and it is an honourable characteristic of modern medicine) that *real talent* has not only been quickly recognized, but warmly supported in the professional world. Let us look to the writings of Currie, of Hamilton, of Philip, of Abernethy, Pearson, Armstrong, Cooper, &c. &c. and see whether these authors were attacked by adversaries sailing on every wind, and accusing them of plagiary, false assumptions, &c. No! From this, then, we are disposed to doubt that "there is something rotten in the constitution of Denmark." And here we shall indulge in a few reflections which may not be entirely useless to the rising generation. In our professional journey through life, we have seen many bye-ways and near-cuts struck out by some of our contemporaries, in order to get to the summit of the hill of fame, before their fellow travellers. One very favourite, and indeed fashionable road may be termed that of *dash and equipage.* This class take up the idea that every thing in this world, and particularly in this metropolis, consists in shew and outside—that talent, erudition, experience, observation, industry, integrity, and other vulgar attributes or qualities, are all nonsense, and that the only essential requisites are, a large house, a fine carriage, and a handsome livery.

Another class set their hearts and souls on "connection." Medical science is all a bugbear with them—every thing is "connection"! Connection is the summum bonum, the alpha and omega, of reputation and wealth. A third class leans on *popularity* as its great basis. In direct violation of the Horatian precept, "Odi profanum vulgus et arceo," they are totally indifferent to the voice of the faculty, and address their orizons to the popular and credulous ear alone—because, in truth, none else will listen to them!

It has been said, and we believe truly, that if a hare and a snail were started to run one hundred miles, the snail would win the race. This last animal creeps slowly on, but straight forward on its course, uninterrupted and unenvied ; but the hare, with all its swiftness, is perpetually curvetting from side to side, and often remeasuring its steps. It is even so with medical adventurers. He who tries to make a quick journey by means of Icarian wings, may chance to have a dip in the Thames, the Serpentine—or the *Fleet !*

*Who* then are destined to arrive at this envied pinnacle of wealth and fame? and *how* are they to get there? The pyramid of medical science, however broad its base, has but a single stone at its summit. Hence it is clear, that of the thousand who are daily climbing up to seat themselves on this apex, almost all must either stop on the road, or fall back to an humbler station. But there is one glory of the sun, another of the moon, and a third of the stars. And so it is in the medical world. At every step from the base to the apex of the pyramid a man may acquire honour, reputation, and even wealth upon the following terms, and with the following requisites.

1st. He must have native talent. In proportion as this forms the basis of the following items, he will rise, sooner or later, on the pyramid of science; but, without it, he must be content to remain on the foundation steps, or ground floor.

2d. He must have industry, first to acquire the rudiments of his profession, and secondly to apply the rudimental knowledge to the actual purposes of life.

3d. He must be ambitious, but not avaricious. Where the object is wealth rather than reputation, farewell science! Let him turn quack at once, and fill his purse at the expence of the lives or health of his fellow creatures.

4th. He must have a dash of enthusiasm in his character. The love of science must be its own reward with him. He must fly to the sick man's chamber, whether it be in the cellar, the garret, or the palace, and study the phænomena of disease, not for the sake of the fee, but for science and the good of his patient. In one word, let him devote the whole energy of his soul to the advancement of his profession, and if he be possessed of talent, no *human* power can ultimately arrest his progress! On the contrary, there is a charm around professional merit, however defenceless, which like that round innocence or beauty, dashes the dagger of envy from the hand of the assassin of character, and tells, with irresistable eloquence, on the hearts of the great, the good, and the wise!

But to return from this digression to the original subject out of which it naturally grew. We confess, that we are unfriendly to medical controversies when carried on with acrimony and personal hostility, which necessarily injure the dignity of science, and degrade the profession. To those controversies too, which relate to *priority of discovery,* we are extremely indifferent; because we conceive, that it is of little or no consequence to the medical republic, who was the inventor of this or that remedy, provided it be a really useful one. The inventors themselves may deem the matter of much more import, and therefore have a right to state their claims to the public at large. But we are very doubtful if medical journals have any right to sit as judges upon these trials; and we think it much more just, as well as prudent, to leave these decisions to the public and to time, which never fail to come at the truth in the long run.

We may be permitted, however, to glance at some practical points in this discussion. The talents and the experience of Dr. Vetch are well known to the profession, and therefore his opinions on all ophthalmic points are entitled to great attention and respect.

"Sir William insists particularly on the distinction betwixt violent vomiting, kept up for eight or ten hours, by emetic tartar, which he proposes, and a constant degree of nausea, which was one of the means of cure, commonly tried by others. The difference may be readily admitted; and I am able to assert, that if far more efficacious means than either are not had recourse to, in the genuine form of the disease, the termination will add to the number of those who have already fallen victims to its ravages, and will soon prove, that innovation may be tried at too great a risk." P. 6.

In respect to the diseased state of the linings of the palpebræ which supervenes on purulent ophthalmia, Dr. V. still asserts, that the scissars are preferable to the knife for its removal, *when an operation is necessary;* but he also asserts, that—

"1st. Of itself the operation, however frequently repeated, is unequal to the cure of the opaque cornea; while, on the other hand, the treatment I adopted in the disease, does not require the aid of an operation in one case out of fifty.

"2d. That the operation, besides being in itself very painful, requires to be indefinitely repeated, and is often followed by inflammation, while the treatment by the properly graduated application of caustic substances, produces neither pain nor inflammation.

"3d. In many cases where a new and white surface has been obtained, after the repeated use of excision, the cornea often remains vascular; a circumstance which never happens when the cure of the membrane lining the eye-lid, has been effected by the action of escharotics, properly applied, the cure of the cornea invariably keeping pace with that of the membrane." P. 20.

Dr. Vetch has brought some heavy charges of misrepresentation against Sir William Adams, which it is highly incumbent on the latter gentleman to rebut if he can. Sir William asserts, that Dr. Vetch has attended the York Hospital for the purpose of seeing his operations; and that previous to this attendance, he [Dr. V.] had never been in the habit of examining the interior of the upper eye-lids. But, mark the plain declaration of the military surgeon.

"On what grounds Sir W. Adams has had the hardiness to advance an assertion *so wholly without foundation*, I am at a loss to conceive. On the examination of, and in the application to, the inner surface of the upper eye-lids, no man can have insisted more strongly than myself. The other part of the assertion which makes me appear at the York Hospital, for the purpose of seeing Sir William Adams, and his new operations, is equally erroneous; *and up to the present hour I have never been in the same room with Sir William Adams, nor seen any case on which he has operated for opake cornea.*"

This passage affords sufficient proof of what we said respecting the injury sustained by medical science in these acrimonious discussions. What must the public think of that profession, whose members will descend to such wilful mis-statements as are here charged against them? One party must be guilty, and therefore the medical public bleeds in the degradation of any of its constituents. We do not indeed say that a man is not to defend himself against slander and misrepresentation; but we do reprehend, with all our force, such statements as call for this defence. It is a sign of a desperate bad cause, particularly in medical philosophy, when sophistry, certificates, and other artificial props are employed to sustain the edifice of facts, or spread the fame of an author or practitioner. They are temporary and contemptible shifts, which the true medical philosopher would scorn to make use of. The force of *truth* is so great, that no earthly power can permanently suppress it; the weakness of *fiction* is such, that all the scaffolding in the world cannot long sustain its mouldering fabric. Every breath of heaven, even the insubstantial and almost fabulous impulse of a sun-beam, shakes it to its centre!

Dr. Vetch, like a British soldier, goes right forward to his enemy, attacking him in front, *vi et armis*. He does not, like the French generals and admirals, make use of any *manœuvres* to get to *windward* of his adversary, or lead him into an ambush. He probably keeps in mind a proverb that is too much neglected—

Omnia vincit veritas, et prevalebit.

---

☞ *In consequence of a great pressure of Review and other Matter, we are obliged to omit our Foreign Selection Department.* Ed.

